# Why we shouldn’t think about percentages or rate loss when it comes to hearing impairment

**DOI:** 10.1016/j.bjorl.2023.03.004

**Published:** 2023-03-21

**Authors:** José Fernando Polanski, Arthur Menino Castilho

**Affiliations:** a Department of Otolaryngology - Head and Neck Surgery, Federal University of Paraná, Curitiba, Brazil; b Mackenzie Evangelical School of Medicine, Curitiba, Brazil; Department of Otolaryngology – Head and Neck Surgery, UNICAMP, Campinas, Brazil

Hearing loss is a sensory deficiency with enormous repercussions on language development, school learning, interpersonal communication, and the psychosocial well-being of individuals. It also has significant economic reflexes on the whole society. A recent publication has brought even more spotlights to the hearing problem: the World Health Organization (WHO) estimates that by 2050, a quarter of the world's population, or 2.5 billion people, will have some degree of hearing loss. In addition, the same report points out that 1.1 billion young people would be at risk of permanent hearing loss due to musical exposure at high and prolonged sound levels^1^. This favorable context for awareness of the hearing issue relives another widely disseminated aspect when it comes to hearing: the questioning as to how much hearing has been lost and how much one can gain from treatment^1^. This human tendency of numerical quantification is understandable and justifiable in order to facilitate understanding and assimilate the facts and concepts. However, it brings inaccuracies.

Occupational rules used in the past in several countries, including Brazil, brought results based on a table score developed in the 1940s in the United States, the Fowler table. This table correlated hearing loss, measured in decibels, with a percentage of difficulty in recognizing speech, indicating a rate of hearing loss^2^. The calculations to reach the final values were quite complex and based on some definitions, such as audiometric “zero”. The audiometric “zero”, or the minimum value of sound perception, was determined in young people with normal hearing in acoustic laboratories and not in normal daily conditions, which certainly adds unfavorable hearing situations, but is real. The defined parameters were based on the English language, which makes it difficult to transpose accurately phonetics into other languages. Another difficulty occurs when more than one side is affected, or even two sides are affected asymmetrically. In these cases, the recommendation was to calculate the weighted mean between the best and worst sides in the 7:1 or 5:1 or 4:1 ratio. In this way, the worst side was almost ignored. This method to define percentages of hearing loss is adequate to determine civil or labor identifications from a pecuniary perspective. Still, it lacks scientific basis^3^.

Another problem related to the percentage determination of hearing loss concerns mathematics. The unit of measurement of the sound pressure level is called Bel, better represented by its tenth part, the decibel (dB), and is defined on a logarithmic scale. Being a logarithmic scale, it has no extremes that can be delimited from the mathematical point of view, presenting virtually infinite values^4^. Thus, it becomes difficult to determine a maximum weight of 100% and a minimum value of 0% from a mathematical perspective. This fact that a logarithmic equation defines it also causes the unit of measurement of sound intensity to have no linear scale, resulting in the fact that, for example, a change of 3 dB doubles the sound intensity. That's why the standard safe sound exposure time is from 8 h to 85 dB and goes to 4 h, or drops in half, by increasing the intensity to 88 dB.

There are methods of establishing a maximum and minimum hearing value based on hearing averages of groups of individuals, known as the minimum hearing threshold and the maximum hearing threshold or pain threshold. With this, it would be possible to establish the maximum or 100% and the minimum or 0%. These standardizations are performed with groups of healthy individuals and supposedly with normal hearing but do not consider anatomical and physiological variability among individuals, and not even equity of conditions, such as atmospheric pressure, temperature, standardization of acoustic isolation, besides the evident subjectivity about which would be a maximum pain threshold. In the normal audiogram, we know that even what is usually defined as a 0 dB threshold, does not always limit the minimum value of auditory perception. Individuals with Superior Semicircular Canal Dehiscence (SSCD) may sometimes present auditory perception at lower thresholds than the 0 dB. Thus, the minimum threshold would not be the minimum for all patients, and the maximum threshold or pain threshold would not be the maximum for all, given the known variability of the pain threshold among individuals^5^.

From the audiological standards, we know that individuals with conductive hearing loss present communication difficulties differently from those with sensorineural losses. Even among those last ones, we know that there are differences between those with cochlear losses from those with retrocochlear diseases. Similarly, individuals with central auditory processing disorders (CAPD) may present various manifestations of hearing disabilities. It may involve problems of recognition and interpretation of sounds and speech and could happen even in situations where the tonal auditory thresholds are normal.

It would still be possible to list here other conditions and factors that make this attempt of quantifying via percentages a failure and susceptible to criticism: the various audible sound frequencies which are not all included in the audiometric thresholds used in pure-tone average; the existence of binaural hearing and age differences.

Thus, we believe that the classification of hearing loss in degrees: mild, moderate, severe, profound, or similar, is the most appropriate classification from the medical and scientific perspective, although not fully able to frame any technical, legal, or even medical situation. Moreover, the attempt to quantify, in percentage, a human sensation, is just an empirical simplification of a much more complex event.
